# Ameliorative effect of lycopene effect on cisplatin-induced nephropathy in patient

**DOI:** 10.15171/jnp.2017.25

**Published:** 2017-01-17

**Authors:** Leila Mahmoodnia, Keivan Mohammadi, Rohollah Masumi

**Affiliations:** Department of Internal Medicine, Shahrekord University of Medical Sciences, Shahrekord, Iran

**Keywords:** Lycopene, Nephrotoxicity, Cisplatin, Cancer

## Abstract

**Background:**

Nephrotoxicity is one of the most important limitations of cisplatin-based chemotherapies which associated with many complications and high mortality rate.

**Objectives:**

To investigate the effect of lycopene on cisplatin-induced nephrotoxicity in patients with cancer.

**Patients and Methods:**

In this double-blind, randomized clinical trial, 120 patients were randomly assigned to two groups, case (treated with lycopene + standard regimen of kidney injury prevention) and control (treated with only the standard regimen of kidney injury prevention). Lycopene was orally taken from 24 hours before to 72 hours after cisplatin administration. Blood urea nitrogen (BUN), serum creatinine (Cr), and glomerular filtration rate (GFR) were measured and recorded. The data were analyzed using SPSS.

**Results:**

Changes in Cr were not significantly different between the two groups (P = 0.131). However, a significant decreasing trend was seen in GFR during the study, which was more marked in the control group (P = 0.004). BUN significantly decreased during the study (P = 0.002), and a significant decrease of BUN on the day three in both groups was seen (P = 0.001). However, BUN increased in the case group on the day 21 of treatment. The corresponding increase was less marked in the control group.

**Conclusions:**

Lycopene can be considered a useful adjuvant therapy to decrease the complications due to cisplatin-induced nephrotoxicity in patients with cancer.

**Implication for health policy/practice/research/medical education:**

Lycopene can be effective in decreasing the complications due to cisplatin-induced nephrotoxicity through affecting some markers of renal function. Therefore, use of lycopene, at appropriate doses, can be considered an adjuvant therapy, alongside chemotherapy and complementary therapy, to decrease the effects of nephrotoxicity.

## 1. Background

Cis-diamminedichloroplatinum(II) [cis-(Pt(NH3)2Cl2), cisplatin)], commonly referred to as CDDP, is a synthetic and anticancer compound (1) which is used depending on the type and histological degree of tumor, disease stage, and the patient’s tolerance to side effects (2). Cisplatin can damage both cancer and healthy cells and nephrotoxicity is one of the complications due to cisplatin (3).

Risk of nephrotoxicity due to cisplatin is an obstacle to using high doses of this drug and hence achieving maximum therapeutic effect. Cisplatin can cause apoptosis of renal proximal tubular cells (LLC-PK1) through activating mitochondrial pathways (4). Therefore, frequent nephrotoxicity is one of the most important limitations of cisplatin-based chemotherapy which is associated with many complications and high mortality rate (5).

Cisplatin-induced nephrotoxicity occurs due to production of reactive oxygen species (ROS), especially hydroxyl radicals, which leads to lipids peroxidation, oxidation of proteins, lipids, and nucleic acids, and destruction of cell membrane. Producing such free radicals causes decline in glomerular filtration rate (GFR), variations in some blood-based biomarkers, and incidence of acute tubular nephrotoxicity. These complications occur mainly in proximal tubes (S3 segment) (6,7). Cisplatin enters into renal epithelial cells and may cause damage to mitochondrial nuclear DNA compounds. Then, it may cause predisposition to apoptosis and necrosis via producing ROS and activating mitochondrial and non-mitochondrial pathways (8).

Carotenoids, including lycopene, are very strong antioxidants and can prevent the toxic effects of other substances in the body (9). Carotenoids can react chemically with ROS and oxidize, and therefore prevent cell damage due to ROS (10). However, simultaneous use of antioxidant compounds and anticancer drugs is still being debated, because excessive use of antioxidant compounds can intervene in chemotherapy and cancer treatment (11). Besides that, no ameliorate impacts have been obtained on the use of antioxidants in some studies (12,13).

Despite oxidative agents play the most important role in renal tissue damage and cisplatin-induced nephrotoxicity (14) and few studies have investigated this issue in human beings. Thus, the role of antioxidants should be investigated in preventing such damages. Some studies are seeking to detect the best protective agent for different tissues.

The role of lycopene has not yet been adequately studied in protecting against cisplatin-induced nephrotoxicity. Besides that, to the best of our knowledge, no similar study has yet been conducted in Iran.

## 2. Objectives

Aim of this study is to investigate the therapeutic and protective effects of lycopene on cisplatin-induced nephrotoxicity in humans.

## 3. Patients and Methods

### 3.1. Patients

In this double-blind, randomized clinical trial, 120 people were selected from the patients referring to the hematology and oncology clinic of the Shahrekord University of Medical Sciences by convenience sampling. The inclusion criteria were considered to be suffering cancer without primary involvement or renal metastasis, being candidate for administration of 70-100 mg/m^2^ cisplatin, 20-80 years old, creatinine (Cr) < 1.5 mg/dL, normal hearing, no underlying nephropathy and the performance status of 0-2 of Karnofsky. Using random allocation software, we assigned the patients randomly to two groups of cases and controls. The control group was received cisplatin and a standard regimen of kidney injury prevention (hydration + magnesium sulfate).

The case group were administered lycopene+ cisplatin alongside a regimen of kidney injury prevention (hydration + magnesium sulfate). Death, allergy to lycopene, hearing loss, significant neuropathy, and metastatic renal involvement were considered as exclusion criteria.

### 3.2. Intervention and biochemical measurements

Appropriate therapeutic doses of cisplatin were intravenously administered to all the patients according to their body mass index and underlying disease(s) (15,16). Any patients of the case group took a lycopene tablet (25 mg) each 12 hours from 24 hours before to 72 hours after cisplatin administration (17). To investigate cisplatin-induced nephrotoxicity, blood urea nitrogen (BUN), serum Cr (18), and GFR (19) were measured.

At the beginning of the study and on the days 7 and 21 after cisplatin administration, 5-cc blood samples of the participants were taken for measurement of BUN and Cr. Then, GFR was calculated with to Crockroft-Gault formula.

### 3.3. Ethical considerations

1) The research followed the tenets of the Declaration of Helsinki; 2) informed consent was obtained, and 3) the research was approved by the ethical committee of Shahrekord University of Medical Sciences (IR.SKUMS.rec.1394.271). Besides that, the study protocol was registered as In the Iranian Registry of Clinical Trials (IRCT2016050427745N1).

### 3.4. Data analysis

The data were analyzed by SPSS 20 software. The quantitative variables were expressed as mean (standard deviation) or median (interquartile range) and qualitative variables as number (%). To compare quantitative variables, repeated measures analysis of variance (ANOVA) was used and to compare qualitative variables, chi-square and, *P* < 0.05 was considered the level of significance.

## 4. Results

Overall, 120 patients participated in this study. Sixty patients, assigned as cases, received lycopene and a standard regimen of kidney injury prevention and the rest, controls, received only the standard regimen of kidney injury prevention. Fifty-eight (48.3%) participants were female. In the control group, 27 (45%) and in the case group, 31 (57.7%) people were female with no significant difference (*P* = 0.465).

The age of the patients in the case and control groups ranged 18-80 and 24-82 (mean: 56.5± 13.3 and 58.5± 12) years, respectively, with no significant difference according to *t* test (*P* = 0.379).

The weight of the patients in the case and control groups ranged 38-93 kg and 42-88 kg (mean: 64± 10.9 and 66.7 ± 10.9), respectively, with no significant difference according to *t* test (*P* = 0.182).

The three renal function parameters, including Cr, GFR, and BUN measurement results, during the study in the two groups are shown in Table 1.

**Table 1:** The results of renal function markers during the study

**Variable**	**Measurement step**	**Control group** **(mean ± SD)**	**Case group** **(mean ± SD)**	**Significance level between the groups**	**Significance level of time effect**	**Significance level of time and group interaction**
Cr mg/dL	Baseline	1.00 ± 0.24	1.05 ± 0.23	0.261	0.011	0.131
	Day 3	1.00 ± 0.22	1.12 ± 0.65	0.416		
	Day 21	1.17 ± 0.51	1.09 ± 0.42	0.402		
GFR cc/min	Baseline	72.06 ± 17.39	66.76 ± 18.75	0.112	<0.001	0.004
	Day 3	71.92 ± 17.17	69.22 ± 20.28	0.447		
	Day 21	65.01 ± 20.77	67.14 ± 20.10	0.587		
BUN mg/dL	Baseline	26.62 ± 9.80	29.29 ± 9.25	0.129	0.002	<0.001
	Day 3	25.55 ± 7.72	16.17 ± 8.97	0.691		
	Day 21	30.74 ± 11.65	26.82 ± 9.72	0.059		

Abbreviations: Cr, creatinine; GFR, glomerular filtration rate; BUN, blood urea nitrogen.

According to repeated measure ANOVA, the changing trend of serum Cr was not significantly different between the two groups (*P* = 0.131), although this trend was significant throughout the study in both groups (*P* = 0.011).

In addition, a significant change in GFR was seen throughout the study (*P* < 0.001) with a significant difference between the two groups (*P* = 0.004). A decreasing trend of GFR was seen in the control group, however, GFR increased in the case group on the first three days and then decreased partially.

A significant change was seen in BUN throughout the study (*P* = 0.002) too. Additionally, a significant decrease in BUN was seen in both groups on the day three (*P* = 0.001), but BUN increased in the case group on the day 21, however, the corresponding increase was less marked in the control group.

## 5. Discussion

In this study, GFR decreased throughout the study in the control group, but increased in the case group within the first three days and then slightly decreased. BUN had a significantly decreasing trend in both groups with a more marked decrease in the case group, maybe due to hydration. Besides that, a significant change was seen in Cr alongside the study with no significant difference between the two groups.

In this regard, a study demonstrated that cisplatin administration to mice is associated with nephrotoxicity, decreases the GFR, and an increase in Cr and plasma urea. This also caused renal dysfunction associated with increased malondialdehyde (MDA) concentration in renal tissue. Atessahin et al demonstrated that administration of lycopene, before and after cisplatin administration, significantly protected kidneys against nephrotoxicity following cisplatin administration and improved the biomarkers in rats (20).

In the study of Dogukan et al, the Cr and plasma urea levels were significantly lower in cisplatin + lycopene, administered group than cisplatin-administered group after the induction of nephrotoxicity. Moreover, lycopene significantly prevented increase in MDA and caused decrease in lipids peroxidation in mice. Finally, they concluded that lycopene plays a protective role against nephrotoxicity after administration of cisplatin (21). In the study of Erman et al, mice were assigned to four groups, control, lycopene, cisplatin, and cisplatin+ lycopene.

Following nephrotoxicity induction, the findings indicated that use of lycopene, after cisplatin administration, caused decrease in serum BUN, Cr, and other measured biomarkers. Thus, lycopene administration significantly resulted to protect kidneys against the complications due to use of cisplatin (22).

A study was conducted to investigate the protective effect of lycopene on ischemic reperfusion renal damage in rats in the short term. After the induction of nephrectomy and perfusion ischemia in rats, the blood samples of rats were investigated for renal biomarkers. Pektas et al found, the pathological score, MDA, glutathione, and catalase of the control group were higher than those of lycopene-treated group. Therefore, lycopene may have protective effects on kidney injuries and related surgeries (23).

In a study on the effect of lycopene on mercuric chloride-induced nephrotoxicity in rats, the findings indicated that lycopene reduced the toxicity due to HgCl(2) through preventing lipids peroxidation and changing delta-aminolevulinate acid dehydratase, and antioxidant enzymes, although it could not prevent the kidney injury induced by HgCl(2) (24).

Overall, antioxidant compounds can decrease the risk of cisplatin-induced nephrotoxicity, which has been confirmed in this study and other researches. Lycopene, an antioxidant, contributes to decreasing oxidative stress and preventing cancer (25). The positive effects of antioxidant compounds have already been demonstrated on cisplatin-induced nephrotoxicity. In a study on the effect of capsaicin on cisplatin-induced nephrotoxicity, use of capsaicin, an antioxidant and anti-inflammatory agent, was found to be effective in decreasing tissue damage and serum levels of BUN and Cr (26).

Ju et al studied the effects of apigenin, a flavonoid, on cisplatin-induced nephrotoxicity and proximal tubular epithelial cells, and found that apigenin caused decrease in P53 activity and therefore decline in apoptosis in renal cells (27). In another study, simvastatin and rosuvastatin was found to be effective in decreasing kidney injuries and lipids peroxidation due to cisplatin (28). A study on the effect of epoxyeicosatrienoic acids on nephrotoxicity and the induction of endoplasmic reticulum stress reported consistent findings, indicating that this compound caused decrease in some blood-based biomarkers such as BUN and Cr through exerting antioxidant effects (29).

However, the study by Mazaheri et al demonstrated that *Foeniculum vulgare* essential oils had no effect on the serum levels of urea and Cr, renal tissue damage, and kidneys weight (13). In addition, a study demonstrated that not only did pomegranate flower extract protect mice against cisplatin-induced nephrotoxicity but it also exacerbated renal tissue damage, although this extract has antioxidant properties (12). This inconsistency can be due to various antioxidant capacities of different substances, lack of extracting effective substances, and the interactions of other substances with treatment course in other studies.

Although antioxidant compounds can exert anti-inflammatory and anti-apoptotic effects on renal cells, the mechanism of cisplatin anti-cancer effect is to induce cell toxicity and cell apoptosis. Excessive use of antioxidants and associated supplements can intervene in cancer treatment, as a review of clinical trials reported that use of antioxidant supplements during chemotherapy and radiotherapy helped to protect tumor and hence reduced survival (11).

Therefore, excessive use of these compounds prevents this drug from acting efficiently and effective doses should be sought out. Use of antioxidants is addressed as an approach to prevent cancer. Fighting oxidative stress and free radicals, antioxidants can exert their preventive effects. As a result, antioxidants should be cautiously used to treat cancer and addressed as adjuvant therapies accompanying main treatments.

## 6. Conclusions

Lycopene can be effective in decreasing the complications due to cisplatin-induced nephrotoxicity through affecting some markers of renal function. Therefore, use of lycopene, at appropriate doses, can be considered an adjuvant therapy, alongside chemotherapy and complementary therapy, to decrease the effects of nephrotoxicity. A limitation of the present study was related to the biomarkers used to diagnose nephrotoxicity. BUN and serum Cr may delay the diagnosis of nephrotoxicity. Therefore, use of other biomarkers seems necessary to diagnose nephrotoxicity early.

More biomarkers should be investigated and necessary standards of sensitivity and speed should be considered in the studies on nephrotoxicity. Furthermore, the effective doses of lycopene in decreasing nephrotoxicity with no drug interactions should be sought out.

## Limitations of the study

This is a retrospective single-center study with a limited proportion of patients. Thus, multi-centric studies on this titles suggests. Moreover, future studies are recommended to compare the effects of different antioxidant compounds on nephrotoxicity, to examine the effects of lycopene on the long-term prognosis in patients, and to investigate potentially unknown interactions.

## Conflicts of interest

The authors declared no competing interests.

## Authors’ contribution

All the authors have contributed towards performing the study and preparation of the manuscript and they all have approved the latest version of the article.

## Acknowledgments

This study was performed in Shahrekord University of Medical Sciences. This study was extracted from internal medicine residential thesis of Keivan Mohammadi (Thesis #1280). The authors sincerely thank all individuals who cooperated with this study.

## Funding/Support

This research project was supported by the deputy of research and technology of Shahrekord University of Medical Sciences (#1394-01-90-2773).
